# Desensitization induces hyporesponsiveness and cell-surface phenotype changes on mouse mast cells

**DOI:** 10.1186/2045-7022-4-S3-P36

**Published:** 2014-07-18

**Authors:** Pedro Giavina-Bianchi, Matthieu Picard, Joana Caiado, Veronica Mezzano, Mariana Castells

**Affiliations:** 1 Harvard Medical School, Division of Rheumatology, Allergy and Immunology, Department of Medicine, BWH, USA

## Background

Rapid drug desensitization (DST) protocols have been developed based on clinical evidence, but in vitro studies are lacking. Understanding the mechanisms involved in the early stages of DST will allow improvements in patients' treatment, overcome unwanted adverse reactions, and identify markers for therapeutic efficacy. The aim of this study is to demonstrate and characterize the induction of hyporesponsiveness in murine mast cells by desensitization and activation, phenotyping the cell surface.

## Method

Mouse bone marrow derived mast cells (BMMCs) sensitized with DNP (2,4-dinitrophenol)-IgE were activated or desensitzed to DNP (1 ng). Desensitization was acheived by sequential doubling doses of DNP every 10 min starting at 1pg. After activation or desensitization, BMMCs were rechallenged by an activating dose of DNP to assess hyporesponsiveness. LAMP1 surface expression and ©¬-hexosaminidase (¥â-Hex) release were used as main outcomes.

## Results

DST inhibited the IgE-mediated degranulation of BMMCs as desensitized cells released 43.3% less ¥â-Hex and showed 73.2% lower LAMP1 surface expression compared to activated BMMCs. A group of desensitized and activated BMMCs were challenged again with an extra dose of 1 ng DNP-HAS and additional release of ¥â-Hex and LAMP1 expresssion were not different from the negative control, regardless whether BMMCs had been previously desensitized or activated. The hyporesponsiveness state of BMMCs induced by DST and activation was not due to mediator depletion as calcium ionophore induced marked release of ¥â-Hex in these cells. Expression of Fc¥åRI, PDL1 and GP49 was assessed next. Activated and desensitized BMMCs, respectively, expressed 40.3% and 15.7% less Fc¥åRI, 45.9% and 13.1% less PDL1, and 23.5% and 11.3% less GP49 than negative controls. We observed that while the expression of PDL1 on the cell membrane decreased, its intracellular amount increased.

## Conclusion

Rapid desensitization and activation induced hyporesponsiveness in murine mast cells, which can be assessed by LAMP1 expression and ¥â-Hex secretion. Hyporesponsiveness is not due to depletion of mediators or mediated by a soluble factors, and is associated with cell-surface phenotype changes.

